# Update on the Role of Cannabinoid Receptors after Ischemic Stroke

**DOI:** 10.1155/2012/824093

**Published:** 2012-04-05

**Authors:** Luciano S. A. Capettini, Silvia Q. Savergnini, Rafaela F. da Silva, Nikos Stergiopulos, Robson A. S. Santos, François Mach, Fabrizio Montecucco

**Affiliations:** ^1^Laboratory of Hemodynamics and Cardiovascular Technology, Institute of Bioengineering, Ecole Polytechnique Federale de Lausanne, BM 5125, Station 17, 1015 Lausanne, Switzerland; ^2^Laboratory of Cardiovascular Pharmacology, Department of Pharmacology, Federal University of Minas Gerais, 31270-901 Belo Horizonte, Brazil; ^3^Laboratory of Hypertension, Department of Physiology and Biophysics, Federal University of Minas Gerais, Belo Horizonte, 31270-901, Brazil; ^4^Division of Cardiology, Foundation for Medical Researches, Department of Medical Specialties, University of Geneva, Avenue de la Roseraie 64, 1211 Geneva, Switzerland

## Abstract

Cannabinoids are considered as key mediators in the pathophysiology of inflammatory diseases, including atherosclerosis. In particular, they have been shown to reduce the ischemic injury after acute cardiovascular events, such as acute myocardial infarction and ischemic stroke. These protective and anti-inflammatory properties on peripheral tissues and circulating inflammatory have been demonstrated to involve their binding with both selective cannabinoid type 1 (CB_1_) and type 2 (CB_2_) transmembrane receptors. On the other hands, the recent discoveries of novel different classes of cannabinoids and receptors have increased the complexity of this system in atherosclerosis. Although only preliminary data have been reported on the activities of novel cannabinoid receptors, several studies have already investigated the role of CB_1_ and CB_2_ receptors in ischemic stroke. While CB_1_ receptor activation has been shown to directly reduce atherosclerotic plaque inflammation, controversial data have been shown on neurotransmission and neuroprotection after stroke. Given its potent anti-inflammatory activities on circulating leukocytes, the CB_2_ activation has been proven to produce protective effects against acute poststroke inflammation. In this paper, we will update evidence on different cannabinoid-triggered avenues to reduce inflammation and neuronal injury in acute ischemic stroke.

## 1. Introduction

Ischemic stroke has become one of the leading causes of mortality and severe disability in several countries, including developing nations [1, 2]. It is provoked by an acute, complete, and prolonged interruption of the arterial flow in the brain characterized by residual tissue infarction [3]. Although extensive studies have been performed to investigate the role of different factors influencing stroke sequelae, the disease pathophysiology remains largely unclear. Physical steps of the ischemic event (such as the transient or permanent interruption of the blood flow and the focal or global cerebral ischemia) are clearly pivotal determinants for the disease prognosis. However, these aspects do not explain some spatial heterogeneity in the cellular damage that might directly reflect neuronal intrinsic susceptibility to injury [4, 5]. Since cannabinoids might accumulate in the ischemic brain [6, 7] and bind their receptors in neurons [8], promising neuroprotective strategies targeting this system to reduce the neuronal ischemic injury have been investigated. On the other hand, since cannabinoids have been shown to modulate brain resident microglial cells [9, 10], cerebral blood vessels [11–14], and circulating inflammatory cells [15, 16], a second therapeutic approach targeting postischemic inflammation has been also explored. In the following paragraphs, we will update scientific results on the role of the cannabinoid receptors as potential regulators of both nervous and immune systems after ischemic stroke [9, 17, 18].

## 2. Cannabinoids and Their Receptors

Endogenous cannabinoids (endocannabinoids) are chemically amides and esters of long polyunsaturated fatty acids including arachidonoylethanolamide (anandamide [AEA]) and 2-arachidonoylglycerol (2-AG). AEA is a minor constituent of the N-acylethanolamines (NAEs) family and has been found elevated in serum and plaques of patients with severe atherosclerotic diseases [19, 20]. On the other, hand, 2-AG has been shown to reach higher concentrations than anandamide analogues (such as palmitoylethanolamide [PEA] and oleoylethanolamide [OEA]) in the brain and atherosclerotic vessels [21]. Synthetic cannabinoids have been also investigated in animal models showing an improvement of the ischemic injury in the liver, heart, and brain [22–24]. Furthermore, phytocannabinoids have been also isolated from the *Cannabis sativa*. Since this plant contains about 80 different cannabinoids, a strong work is still needed to test all these active compounds. This delay in cannabinoid research might be also due to the very low dose of certain cannabinoids in the plant. Thus, since Δ^9^-tetrahydrocannabidiol (THC) and cannabidiol (CBD) represent up to 40% of the total cannabinoid mass [25], these compounds have been considered as the most active mediators.

The effects of cannabinoids are classically attributed to the activation of the two major cannabinoids receptors (the cannabinoid receptors type 1 [CB_1_] and type 2 [CB_2_]). These transmembrane receptors are pivotal components of the physiological endocannabinoid system together with endogenous cannabinoids (endocannabinoids), their transporters, and synthetic/degrading enzymes [26]. CB_1_ is highly expressed in the brain [27–31] as well as in cardiovascular tissues including the heart [32–34] and vascular vessels [35–38]. Although it has been recently shown in the brain [39, 40], CB_2_ is mainly expressed in the immune and hematopoietic cells [10, 41–43]. Importantly, novel receptors capable of binding cannabinoids have been recently identified. In particular, the transient receptor potential vanilloid (TRPV) channels might be activated by cannabinoids [44–46]. Among these channels, the activity and expression of TRPV type 1, 3, and 4 could be modulated by classical cannabinoids receptors agonists [41, 47–52]. On the other hand, there is growing evidence that cannabinoids can activate the peroxisome proliferator-activated receptors (PPAR, a family of nuclear receptors) in neurological diseases [53–57]. Although its activity has not been clarified yet, the orphan receptor GPR55 has been also identified to potentially bind cannabinoids and intracellularly transduce their signals [58, 59].

## 3. Role of Cannabinoid Receptors in Poststroke Inflammatory Injury

Some key events in the pathophysiology of ischemic stroke include increased levels of inflammatory cytokines in the brain, activation of microglia, and adhesion and migration of peripheral leukocytes as a result of damage to the blood-brain barrier [60]. A severe immunosuppression also characterized by spleen atrophy has been described to follow this initial inflammatory burst [61]. In order to inhibit the acute inflammatory phase (strongly associated with cerebral injury) [62], the treatment with Δ^9^-tetrahydrocannabinol (THC, a cannabinoid receptor agonist) has been shown to induce immunomodulatory properties *in vitro* [63–65]. Endocannabinoids (such as AEA and 2-AG) might be also released by immune cells and neurons, thus locally modulating immune response and cell differentiation within the brain [66, 67]. Several immune cells (such as lymphocytes, monocytes, and neutrophils, capable of infiltrating the injured brain) have been shown to express on their surface membrane both CB_1_ and CB_2_ receptors [41, 47, 68–71]. CB_1_ receptor is particularly expressed on T lymphocytes and might be further upregulated by cannabinoid stimulation [69, 72–75]. This mechanism might favor the paracrine protective activity of AEA, which is highly produced in the ischemic brain area and locally inhibits T lymphocyte proliferation [76]. Importantly, CB_1_ expression has been also confirmed on cerebral macrophage-like cells, suggesting a potential direct effects of cannabinoids also on populations resident in the brain [77]. On the other hand, the majority of beneficial effects of cannabinoids is associated with CB_2_ receptor activation, which is classically described to inhibit immune proinflammatory functions. CB_2_ receptor is also expressed in immune organs (such as thymus and spleen) [78] and circulating inflammatory cells (including T-, B-lymphocytes, NK cells and monocytes and neutrophils) [79–81]. Importantly, CB_2_ protein has been recently detected in astrocytes [82], microglia [83], neural subpopulations, and oligodendroglial progenitors [84], suggesting a potential direct regulation of CB_2_ in cerebral poststroke inflammation also in brain inflammatory cells [85]. The levels of CB_2_ receptor on microglial cells might depend on the cell activation state in response to infection, inflammation and stress [66, 86]. CB_2_ receptor is also upregulated in restricted areas of the spinal cord in response to peripheral nerve injury [87], suggesting that neurons and resident inflammatory cells might benefit of cannabinoid treatments. Since CB_1_ and CB_2_ surface expression is upregulated during the inflammatory activation [66, 88–90], treatment with cannabinoids might be even more effective in the early post-ischemic phase. Treatment with the selective CB_2_ agonist JWH-133 was shown to significantly reduce microglial activation and inflammatory gene expression (such as interleukin [IL]-6, tumor necrosis factor [TNF]-*α*, regulated on activation, normal T-cell expressed and secreted [RANTES], monocyte chemoattractant protein [MCP]-1, and macrophage inflammatory peptide [MIP]-1*α*) in a mouse model of permanent middle cerebral artery occlusion and focal cerebral ischemia [91]. Importantly, this potent anti-inflammatory activity was accompanied by the JWH-133-mediated improvement of brain infarction and neurological “clinical” outcomes [91]. Accordingly, pretreatment with CB_2_ agonists has been shown to attenuate the poststroke enhancement of leukocyte/endothelial cells interaction, adhesion molecule expression, and disruption of blood-brain barrier (BBB) [92–94]. In addition, CB_2_ knockout mice developed an increased cerebral infarction, accompanied by worsened neurological functions when compared to wild-type mice [92].

Confirming recent studies in other macrophage-mediated inflammatory diseases (such as atherosclerosis and rheumatoid arthritis) [15, 18, 95], these studies clearly show that CB_2_ activation might actively reduce the post-stroke cerebral microglial inflammation.

Cannabinoids might also affect T lymphocyte function and survival [65]. Although the role of T lymphocytes in post-stroke inflammation has not been clarified yet in human beings [96, 97], recent evidence from mouse models indicated that these cells might modulate their functional capacities after an acute cerebral ischemia [61, 62, 98, 99]. In particular, the CD4+CD25+ regulatory T cells have been suggested to play cerebroprotective activities in mice after a focal cerebral ischemia [62, 98]. Therefore, the cannabinoid-mediated benefits might involve the T lymphocyte response. In other inflammatory diseases, cannabinoid agonism has been shown to affect proliferation, polarization [66, 100], and cytolytic capacity [101] of T cells. Recently, Tanikawa and coworkers published that treatment with WIN-55,212-2 favors the lymphocyte migration within the spleen, supporting that this cannabinoid agonist directly downregulates the immune response [102]. Accordingly, 2-AG strongly reduces mitogen-induced proliferation of mouse splenocytes [103]. These studies support a potential therapeutic role of cannabinoid agonists to reduce T lymphocyte-mediated inflammation in the post-stroke acute phase.

Although some effort has been made in the past decades to clarify the role of the cannabinoid system in post-stroke inflammation, discrepancies deriving from the use of different types and doses of cannabinoids in both *in vivo* and *in vitro* models still exist and represent a major limitation to define clear conclusions.

## 4. The Cannabinoid System in the Ischemic Neuronal Injury

The acute reduction of blood flow with the consequent abrogation of oxygen and nutrient supply in the peripheral cerebral tissue has been shown to significantly modify the neural electrolytic equilibrium and metabolism. The cytosolic increase of reactive oxygen species (ROS), calcium, and sodium has been described to directly increase neuron necrosis and apoptosis in the early hours after the ischemic onset [104]. Normally, the burst of ROS in the cytosol is accompanied by a reduction in pH, while the release of glutamate from the core of the infarcted area increases the permanency of high intracellular calcium concentrations, thus contributing to neuron injury. Deprivation of oxygen and glucose during ischemia also contributes to the increase in cytosolic Ca^2+^ via NMDA receptor-mediated pathways [105]. Since drugs blocking Ca^2+^ channels have been shown to protect cells, Ca^2+^ influx is considered as pivotal pathophysiological mechanism during an ischemic insult [106]. Together with the acute leukocyte influx, these neuronal modifications are probably the most relevant events in the pathophysiology of cerebral ischemia/reperfusion [107, 108].

Thus, cannabinoid-related mechanisms might serve as promising candidates for the reduction of neuronal ischemic injury. In particular, AEA and 2-AG have been shown to especially accumulate in the brain ischemic areas [13, 109]. This enhancement occurs exponentially in a time-dependent manner [110], suggesting a potential protective role for endocannabinoids against the neuronal ischemic injury. However, several discrepancies on the activation of different cannabinoid receptors in experimental models of cerebral ischemia have to be clarified. In particular, the activation of CB_1_ receptor has been firstly indicated as a neuroprotective strategy in transient cerebral ischemia models [111]. On the other hand, Pegorini and coworkers also showed that treatment with rimonabant (a selective CB_1_ antagonist but also a VR1 vanilloid receptor agonist) might increase neuroprotection via the activation of this ligand-gated cation channel [112]. These apparently paradoxical results may find an explanation in the different affinities of cannabinoids for their receptors. In fact, depending on the concentrations, cannabinoids might act as both agonists and antagonists for certain receptors in the same time. Importantly, an increase in CB_2_ receptor and TRPV1 and a concomitant decrease in CB_1_ cerebral expression were observed in mice underwent permanent middle cerebral artery occlusion and intraperitoneally injected with leptin [113]. The improvements of residual neurological disability were associated with reduced infarct volume in brain, suggesting that these receptors might beneficially influence the neuronal ischemic injury [113].

Taking into account these premises, the cannabinoid receptors have been shown to activate defined intracellular pathways in ischemic neurons [111, 114]. Specifically, CB_1_ receptor activation triggers several protective signals involving phosphorylation of mitogen-activated protein kinase (MAPK) kinase 1/2 (MEK1/2), extracellular signal-regulated kinase (ERK1/2), and nuclear factor-kappa B (NF-*κ*B) [114, 115]. These intracellular pathways might increase neuronal survival. The first-in-man study, investigating the cannabinoid therapeutic approach on neuroprotection, showed that the intravenous infusion of THC ameliorates the global and regional cerebral blood flow up to 2 h after the infusion of both low (0.15 mg/min) and high (0.25 mg/min) doses [116]. The encouraging therapeutic results of this study are in partial contrast with previous case reports, suggesting a potential relationship between stroke and chronic cannabis abuse in young human beings [117–120]. Importantly, Mateo and coworkers recently confirmed the potential association between cannabis and ischemic stroke recurrence in a young patient, without identifying the underlying pathophysiological mechanisms [121]. Evidence from autopsy examinations and imaging reports in both human beings and animal models has suggested that cannabis use might provoke cerebral stroke by favouring the development of atrial fibrillation, orthostatic hypotension, and cerebral artery vasospasm [122–124]. In a prospective study enrolling 48 young adults affected by ischemic stroke, Wolff and co-workers also showed that multifocal intracranial arterial stenosis was associated with cannabis consumption in 21% [125]. Therefore, these preliminary results in human beings suggest using caution in the translational therapeutic approach of cannabinoid from animal models. Further clinical studies (possibly using more selective cannabinoid receptor agonist/antagonist) are needed to clarify the potential therapeutic role of these compounds against the ischemic stroke.

## 5. Conclusion

The endocannabinoid system is considered as a major modulator of the cerebral blood flow, neuroinflammation, and neuronal survival. Despite of some controversies, the activation of CB_2_ receptor has been shown to reduce cerebral injury associated with acute post-stroke inflammation and leukocyte infiltration. On the other hand, the direct role of CB_1_ in neuronal protection has not been clarified yet. Evidence from animal models and *in vitro* studies suggests a global protective role for cannabinoid receptors agonists in ischemic stroke. However, further studies are needed to clarify the role of the recently discovered cannabinoid receptors (such as GPR55 or VR1 vanilloid receptor) in the physiopathology of the infarcted brain and related inflammation. At this regard, both synthetic cannabinoids and endocannabinoids represent extremely promising therapeutic compounds. Since human studies are still missing, we cannot predict the potential clinical benefits of treatments targeting cannabinoid receptors in ischemic stroke. We believe that the “cannabinoid” approach represents an interesting therapeutic strategy still requiring further validations to improve neurologic and inflammatory outcomes in ischemic stroke.

## References

[1] Egan RA, Biousse V (2000). Update on ischemic stroke. *Current Opinion in Ophthalmology*.

[2] Stankovic S, Majkic-Singh N (2010). Genetic aspects of ischemic stroke: coagulation, homocysteine, and lipoprotein metabolism as potential risk factors. *Critical Reviews in Clinical Laboratory Sciences*.

[3] Easton JD, Saver JL, Albers GW (2009). American Heart Association; American Stroke Association Stroke Council; Council on Cardiovascular Surgery and Anesthesia; Council on Cardiovascular Radiology and Intervention; Council on Cardiovascular Nursing; Interdisciplinary Council on Peripheral Vascular Disease. Definition and evaluation of transient ischemic attack: a scientific statement for healthcare professionals from the American Heart Association/American Stroke Association Stroke Council; Council on Cardiovascular Surgery and Anesthesia; Council on Cardiovascular Radiology and Intervention; Council on Cardiovascular Nursing; and the Interdisciplinary Council on Peripheral Vascular Disease. The American Academy of Neurology affirms the value of this statement as an educational tool for neurologists. *Stroke*.

[4] Lipton P (1999). Ischemic cell death in brain neurons. *Physiological Reviews*.

[5] Vaughn LK, Denning G, Stuhr KL, De Wit H, Hill MN, Hillard CJ (2010). Endocannabinoid signalling: has it got rhythm?. *British Journal of Pharmacology*.

[6] Hillard CJ, Jarrahian A (2005). Accumulation of anandamide: evidence for cellular diversity. *Neuropharmacology*.

[7] Pryce G, Ahmed Z, Hankey DJR (2003). Cannabinoids inhibit neurodegeneration in models of multiple sclerosis. *Brain*.

[8] Shen M, Piser TM, Seybold VS, Thayer SA (1996). Cannabinoid receptor agonists inhibit glutamatergic synaptic transmission in rat hippocampal cultures. *Journal of Neuroscience*.

[9] Carrier EJ, Patel S, Hillard CJ (2005). Endocannabinoids in neuroimmunology and stress. *Current Drug Targets*.

[10] Maresz K, Carrier EJ, Ponomarev ED, Hillard CJ, Dittel BN (2005). Modulation of the cannabinoid CB_2_ receptor in microglial cells in response to inflammatory stimuli. *Journal of Neurochemistry*.

[11] Chen Y, McCarron RM, Ohara Y (2000). Human brain capillary endotheliumml: 2-Arachidonoglycerol (endocannabinoid) interacts with endothelin-1. *Circulation Research*.

[12] Gebremedhin D, Lange AR, Campbell WB, Hillard CJ, Harder DR (1999). Cannabinoid CB_1_ receptor of cat cerebral arterial muscle functions to inhibit L-type Ca2+ channel current. *American Journal of Physiology*.

[13] Hillard CJ, Ho WSV, Thompson J (2007). Inhibition of 2-arachidonoylglycerol catabolism modulates vasoconstriction of rat middle cerebral artery by the thromboxane mimetic, U-46619. *British Journal of Pharmacology*.

[14] Wagner JA, Járai Z, Bátkai S, Kunos G (2001). Hemodynamic effects of cannabinoids: coronary and cerebral vasodilation mediated by cannabinoid CB_1_ receptors. *European Journal of Pharmacology*.

[15] Montecucco F, Burger F, Mach F, Steffens S (2008). CB_2_ cannabinoid receptor agonist JWH-015 modulates human monocyte migration through defined intracellular signaling pathways. *American Journal of Physiology*.

[16] Montecucco F, Lenglet S, Braunersreuther V (2009). CB_2_ cannabinoid receptor activation is cardioprotective in a mouse model of ischemia/reperfusion. *Journal of Molecular and Cellular Cardiology*.

[17] Ashton JC (2007). Cannabinoids for the treatment of inflammation. *Current Opinion in Investigational Drugs*.

[18] Steffens S, Veillard NR, Arnaud C (2005). Low dose oral cannabinoid therapy reduces progression of atherosclerosis in mice. *Nature*.

[19] Quercioli A, Pataky Z, Vincenti G (2011). Elevated endocannabinoid plasma levels are associated with coronary circulatory dysfunction in obesity. *European Heart Journal*.

[20] Montecucco F, Di Marzo V, da Silva RF (2012). The activation of the cannabinoid receptor type 2 (CB_2_) reduces neutrophilic protease-mediated vulnerability in atherosclerotic plaques. *European Heart Journal*.

[21] Hillard CJ (2008). Role of cannabinoids and endocannabinoids in cerebral Ischemia. *Current Pharmaceutical Design*.

[22] De Petrocellis L, Vellani V, Schiano-Moriello A (2008). Plant-derived cannabinoids modulate the activity of transient receptor potential channels of ankyrin type-1 and melastatin type-8. *Journal of Pharmacology and Experimental Therapeutics*.

[23] Downer EJ, Campbell VA (2010). Phytocannabinoids, CNS cells and development: a dead issue?. *Drug and Alcohol Review*.

[24] Pacher P, Haskó G (2008). Endocannabinoids and cannabinoid receptors in ischaemia-reperfusion injury and preconditioning. *British Journal of Pharmacology*.

[25] Gertsch J, Pertwee RG, Di Marzo V (2010). Phytocannabinoids beyond the Cannabis plant—do they exist?. *British Journal of Pharmacology*.

[26] Vettor R, Pagano C (2009). The role of the endocannabinoid system in lipogenesis and fatty acid metabolism. *Best Practice and Research*.

[27] Aguado T, Romero E, Monory K (2007). The CB_1_ cannabinoid receptor mediates excitotoxicity-induced neural progenitor proliferation and neurogenesis. *Journal of Biological Chemistry*.

[28] Ashton JC, Appleton I, Darlington CL, Smith PF (2004). Immunohistochemical localization of cerebrovascular cannabinoid CB_1_ receptor protein. *Journal of Cardiovascular Pharmacology*.

[29] Benito C, Kim WK, Chavarría I (2005). A glial endogenous cannabinoid system is upregulated in the brains of macaques with simian immunodeficiency virus-induced encephalitis. *Journal of Neuroscience*.

[30] Hohmann AG, Herkenham M (1999). Localization of central cannabinoid CB_1_ receptor messenger RNA in neuronal subpopulations of rat dorsal root ganglia: a double-label in situ hybridization study. *Neuroscience*.

[31] Smith TH, Sim-Selley LJ, Selley DE (2010). Cannabinoid CB_1_ receptor-interacting proteins: novel targets for central nervous system drug discovery?. *British Journal of Pharmacology*.

[32] Bátkai S, Mukhopadhyay P, Harvey-White J, Kechrid R, Pacher P, Kunos G (2007). Endocannabinoids acting at CB_1_ receptors mediate the cardiac contractile dysfunction in vivo in cirrhotic rats. *American Journal of Physiology*.

[33] Howlett AC, Blume LC, Dalton GD (2010). CB_1_ cannabinoid receptors and their associated proteins. *Current Medicinal Chemistry*.

[34] Mukhopadhyay P, Rajesh M, Bátkai S (2010). CB_1_ cannabinoid receptors promote oxidative stress and cell death in murine models of doxorubicin-induced cardiomyopathy and in human cardiomyocytes. *Cardiovascular Research*.

[35] Bátkai S, Járai Z, Wagner JA (2001). Endocannabinoids acting at vascular CB_1_ receptors mediate the vasodilated state in advanced liver cirrhosis. *Nature Medicine*.

[36] Chaytor AT, Martin PEM, Evans WH, Randall MD, Griffith TM (1999). The endothelial component of cannabinoid-induced relaxation in rabbit mesenteric artery depends on gap junctional communication. *Journal of Physiology*.

[37] Rajesh M, Mukhopadhyay P, Haskó G, Liaudet L, MacKie K, Pacher P (2010). Cannabinoid-1 receptor activation induces reactive oxygen species-dependent and -independent mitogen-activated protein kinase activation and cell death in human coronary artery endothelial cells. *British Journal of Pharmacology*.

[38] Tiyerili V, Zimmer S, Jung S (2010). CB_1_ receptor inhibition leads to decreased vascular AT1 receptor expression, inhibition of oxidative stress and improved endothelial function. *Basic Research in Cardiology*.

[39] Núñez E, Benito C, Pazos MR (2004). Cannabinoid CB_2_ receptors are expressed by perivascular microglial cells in the human brain: an Immunohistochemical Study. *Synapse*.

[40] Palazuelos J, Aguado T, Egia A, Mechoulam R, Guzmán M, Galve-Roperh I (2006). Non-psychoactive CB_2_ cannabinoid agonists stimulate neural progenitor proliferation.. *The FASEB Journal*.

[41] Atwood BK, MacKie K (2010). CB_2_: a cannabinoid receptor with an identity crisis. *British Journal of Pharmacology*.

[42] Kasten KR, Tschöp J, Tschöp MH, Caldwell CC (2010). The cannabinoid 2 receptor as a potential therapeutic target for sepsis. *Endocrine, Metabolic and Immune Disorders*.

[43] Ni X, Geller EB, Eppihimer MJ, Eisenstein TK, Adler MW, Tuma RF (2004). Win 55212-2, a cannabinoid receptor agonist, attenuates leukocyte/endothelial interactions in an experimental autoimmune encephalomyelitis model. *Multiple Sclerosis*.

[44] Akerman S, Kaube H, Goadsby PJ (2004). Anandamide acts as a vasodilator of dural blood vessels in vivo by activating TRPV1 receptors. *British Journal of Pharmacology*.

[45] Baker CL, McDougall JJ (2004). The cannabinomimetic arachidonyl-2-chloroethylamide (ACEA) acts on capsaicin-sensitive TRPV1 receptors but not cannabinoid receptors in rat joints. *British Journal of Pharmacology*.

[46] Dannert MT, Alsasua A, Herradon E, Martín MI, López-Miranda V (2007). Vasorelaxant effect of Win 55,212-2 in rat aorta: new mechanisms involved. *Vascular Pharmacology*.

[47] Campos AC, Guimarães FS (2009). Evidence for a potential role for TRPV1 receptors in the dorsolateral periaqueductal gray in the attenuation of the anxiolytic effects of cannabinoids. *Progress in Neuro-Psychopharmacology and Biological Psychiatry*.

[48] Herradón E, Martín MI, López-Miranda V (2007). Characterization of the vasorelaxant mechanisms of the endocannabinoid anandamide in rat aorta. *British Journal of Pharmacology*.

[49] Moezi L, Gaskari SA, Liu H, Baik SK, Dehpour AR, Lee SS (2006). Anandamide mediates hyperdynamic circulation in cirrhotic rats via CB_1_ and VR1 receptors. *British Journal of Pharmacology*.

[50] De Petrocellis L, Di Marzo V (2010). Non-CB_1_, Non-CB_2_ receptors for endocannabinoids, plant cannabinoids, and synthetic cannabimimetics: focus on G-protein-coupled receptors and transient receptor potential channels. *Journal of Neuroimmune Pharmacology*.

[51] de Petrocellis L, Orlando P, Moriello AS (2012). Cannabinoid actions at TRPV channels: effects on TRPV3 and TRPV4 and their potential relevance to gastrointestinal inflammation. *Acta Physiologica*.

[52] Di Marzo V, De Petrocellis L (2010). Endocannabinoids as regulators of transient receptor potential (TRP) channels: a further opportunity to develop new endocannabinoid-based therapeutic drugs. *Current Medicinal Chemistry*.

[53] Burstein S (2005). PPAR-*γ*: a nuclear receptor with affinity for cannabinoids. *Life Sciences*.

[54] Fu J, Gaetani S, Oveisi F (2003). Oleylethanolamide regulates feeding and body weight through activation of the nuclear receptor PPAR-*α*. *Nature*.

[55] O’Sullivan SE, Kendall DA (2010). Cannabinoid activation of peroxisome proliferator-activated receptors: potential for modulation of inflammatory disease. *Immunobiology*.

[56] O'Sullivan SE, Tarling EJ, Bennett AJ, Kendall DA, Randall MD (2005). Novel time-dependent vascular actions of Δ9-tetrahydrocannabinol mediated by peroxisome proliferator-activated receptor gamma. *Biochemical and Biophysical Research Communications*.

[57] Rockwell CE, Snider NT, Thompson JT, Vanden Heuvel JP, Kaminski NE (2006). Interleukin-2 suppression by 2-arachidonyl glycerol is mediated through peroxisome proliferator-activated receptor *γ* independently of cannabinoid receptors 1 and 2. *Molecular Pharmacology*.

[58] Brusberg M, Arvidsson S, Kang D, Larsson H, Lindström E, Martinez V (2009). CB_1_ receptors mediate the analgesic effects of cannabinoids on colorectal distension-induced visceral pain in rodents. *Journal of Neuroscience*.

[59] Irving A (2011). New blood brothers: the GPR55 and CB_2_ partnership. *Cell Research*.

[60] Dirnagl U, Iadecola C, Moskowitz MA (1999). Pathobiology of ischaemic stroke: an integrated view. *Trends in Neurosciences*.

[61] Dziennis S, Mader S, Akiyoshi K (2011). Therapy with recombinant T-cell receptor ligand reduces infarct size and infiltrating inflammatory cells in brain after middle cerebral artery occlusion in mice. *Metabolic Brain Disease*.

[62] Ren X, Akiyoshi K, Vandenbark AA, Hurn PD, Offner H (2011). CD4+FoxP3+ regulatory T-cells in cerebral ischemic stroke. *Metabolic Brain Disease*.

[63] Srivastava MD, Srivastava BIS, Brouhard B (1998). Δ9 Tetrahydrocannabinol and cannabidiol alter cytokine production by human immune cells. *Immunopharmacology*.

[64] Yuan M, Kiertscher SM, Cheng Q, Zoumalan R, Tashkin DP, Roth MD (2002). Δ9-Tetrahydrocannabinol regulates Th1/Th2 cytokine balance in activated human T cells. *Journal of Neuroimmunology*.

[65] Zhu LX, Sharma S, Stolina M (2000). Δ-9-tetrahydrocannabinol inhibits antitumor immunity by a CB_2_ receptor-mediated, cytokine-dependent pathway. *Journal of Immunology*.

[66] Croxford JL, Yamamura T (2005). Cannabinoids and the immune systemml: potential for the treatment of inflammatory diseases?. *Journal of Neuroimmunology*.

[67] Massi P, Vaccani A, Parolaro D (2006). Cannabinoids, immune system and cytokine network. *Current Pharmaceutical Design*.

[68] Mouslech Z, Valla V (2009). Endocannabinoid systemml: an overview of its potential in current medical practice. *Neuroendocrinology Letters*.

[69] Rossi B, Zenaro E, Angiari S (2011). Inverse agonism of cannabinoid CB_1_ receptor blocks the adhesion of encephalitogenic T cells in inflamed brain venules by a protein kinase A-dependent mechanism. *Journal of Neuroimmunology*.

[70] Stefano GB, Bilfinger TV, Rialas CM, Deutsch DG (2000). 2-arachidonyl-glycerol stimulates nitric oxide release from human immune and vascular tissues and invertebrate immunocytes by cannabinoid receptor 1. *Pharmacological Research*.

[71] Steffens S, Mach F (2006). Towards a therapeutic use of selective CB_2_ cannabinoid receptor ligands for atherosclerosis. *Future Cardiology*.

[72] Kaplan BLF, Springs AEB, Kaminski NE (2008). The profile of immune modulation by cannabidiol (CBD) involves deregulation of nuclear factor of activated T cells (NFAT). *Biochemical Pharmacology*.

[73] Mnich SJ, Hiebsch RR, Huff RM, Muthian S (2010). Anti-inflammatory properties of CB_1_-receptor antagonist involves *β*2 adrenoceptors. *Journal of Pharmacology and Experimental Therapeutics*.

[74] Newton CA, Chou PJ, Perkins I, Klein TW (2009). CB_1_ and CB_2_ cannabinoid receptors mediate different aspects of delta-9-tetrahydrocannabinol (THC)-induced T helper cell shift following immune activation by legionella pneumophila infection. *Journal of Neuroimmune Pharmacology*.

[75] Börner C, Höllt V, Kraus J (2007). Activation of human T cells induces upregulation of cannabinoid receptor type 1 transcription. *NeuroImmunoModulation*.

[76] Schwarz H, Blanco FJ, Lotz M (1994). Anadamide, an endogenous cannabinoid receptor agonist inhibits lymphocyte proliferation and induces apoptosis. *Journal of Neuroimmunology*.

[77] Sinha D, Bonner TI, Bhat NR, Matsuda LA (1998). Expression of the CB_1_ cannabinoid receptor in macrophage-like cells from brain tissue: immunochemical characterization by fusion protein antibodies. *Journal of Neuroimmunology*.

[78] Schatz AR, Lee M, Condie RB, Pulaski JT, Kaminski NE (1997). Cannabinoid receptors CB_1_ and CB_2_: a characterization of expression and adenylate cyclase modulation within the immune system. *Toxicology and Applied Pharmacology*.

[79] Carayon P, Marchand J, Dussossoy D (1998). Modulation and functional involvement of CB_2_ peripheral cannabinoid receptors during B-cell differentiation. *Blood*.

[80] Galiegue S, Mary S, Marchand J (1995). Expression of central and peripheral cannabinoid receptors in human immune tissues and leukocyte subpopulations. *European Journal of Biochemistry*.

[81] Mestre L, Docagne F, Correa F (2009). A cannabinoid agonist interferes with the progression of a chronic model of multiple sclerosis by downregulating adhesion molecules. *Molecular and Cellular Neuroscience*.

[82] Stella N (2010). Cannabinoid and cannabinoid-like receptors in microglia, astrocytes, and astrocytomas. *Glia*.

[83] Núñez E, Benito C, Tolón RM, Hillard CJ, Griffin WST, Romero J (2008). Glial expression of cannabinoid CB_2_ receptors and fatty acid amide hydrolase are beta amyloid-linked events in Down’s syndrome. *Neuroscience*.

[84] Onaivi ES (2007). Neuropsychobiological evidence for the functional presence and expression of cannabinoid CB_2_ receptors in the brain. *Neuropsychobiology*.

[85] Ashton JC, Glass M (2007). The cannabinoid CB_2_ receptor as a target for inflammation-dependent neurodegeneration. *Current Neuropharmacology*.

[86] Carlisle SJ, Marciano-Cabral F, Staab A, Ludwick C, Cabral GA (2002). Differential expression of the CB_2_ cannabinoid receptor by rodent macrophages and macrophage-like cells in relation to cell activation. *International Immunopharmacology*.

[87] Zhang J, Hoffert C, Vu HK, Groblewski T, Ahmad S, O’Donnell D (2003). Induction of CB_2_ receptor expression in the rat spinal cord of neuropathic but not inflammatory chronic pain models. *European Journal of Neuroscience*.

[88] Maresz K, Pryce G, Ponomarev ED (2007). Direct suppression of CNS autoimmune inflammation via the cannabinoid receptor CB_1_ on neurons and CB_2_ on autoreactive T cells. *Nature Medicine*.

[89] Klein TW, Newton C, Zhu W, Daaka Y, Friedman H (1995). Δ9-tetrahydrocannabinol, cytokines, and immunity to Legionella pneumophila. *Proceedings of the Society for Experimental Biology and Medicine*.

[90] Daaka Y, Friedman H, Klein TW (1996). Cannabinoid receptor proteins are increased in Jurkat, human T-cell line after mitogen activation. *Journal of Pharmacology and Experimental Therapeutics*.

[91] Zarruk JG, Fernández-López D, García-Yébenes I (2012). Cannabinoid type 2 receptor activation downregulates stroke-induced classic and alternative brain macrophage/microglial activation concomitant to neuroprotection. *Stroke*.

[92] Zhang M, Adler MW, Abood ME, Ganea D, Jallo J, Tuma RF (2009). CB_2_ receptor activation attenuates microcirculatory dysfunction during cerebral ischemic/reperfusion injury. *Microvascular Research*.

[93] Zhang M, Martin BR, Adler MW, Razdan RK, Jallo JI, Tuma RF (2007). Cannabinoid CB_2_ receptor activation decreases cerebral infarction in a mouse focal ischemia/reperfusion model. *Journal of Cerebral Blood Flow and Metabolism*.

[94] Zhao Y, Yuan Z, Liu Y (2010). Activation of cannabinoid CB_2_ receptor ameliorates atherosclerosis associated with suppression of adhesion molecules. *Journal of Cardiovascular Pharmacology*.

[95] Zurier RB, Rossetti RG, Lane JH, Goldberg JM, Hunter SA, Burstein SH (1998). Dimethylheptyl-THC-11 OIC acid: a nonpsychoactive antiinflammatory agent with a cannabinoid template structure. *Arthritis and Rheumatism*.

[96] Haeusler KG, Schmidt WUH, Foehring F (2012). Immune responses after acute ischemic stroke or myocardial infarction. *International Journal of Cardiology*.

[97] Hendrix S, Nitsch R (2007). The role of T helper cells in neuroprotection and regeneration. *Journal of Neuroimmunology*.

[98] Hug A, Liesz A, Muerle B (2011). Reduced efficacy of circulating costimulatory cells after focal cerebral ischemia. *Stroke*.

[99] Yilmaz G, Arumugam TV, Stokes KY, Granger DN (2006). Role of T lymphocytes and interferon-*γ* in ischemic stroke. *Circulation*.

[100] Hegde VL, Hegde S, Cravatt BF, Hofseth LJ, Nagarkatti M, Nagarkatti PS (2008). Attenuation of experimental autoimmune hepatitis by exogenous and endogenous cannabinoids: involvement of regulatory T cells. *Molecular Pharmacology*.

[101] Wacnik PW, Luhr KM, Hill RH, Ljunggren HG, Kristensson K, Svensson M (2008). Cannabinoids affect dendritic cell (DC) potassium channel function and modulate DC T cell stimulatory capacity. *Journal of Immunology*.

[102] Tanikawa T, Kurohane K, Imai Y (2011). Regulatory effect of cannabinoid receptor agonist on chemokine-induced lymphocyte chemotaxis. *Biological and Pharmaceutical Bulletin*.

[103] Lee M, Kyu Hwan Yang, Kaminski NE (1995). Effects of putative cannabinoid receptor ligands, anandamide and 2- arachidonyl-glycerol, on immune function in B6C3F1 mouse splenocytes. *Journal of Pharmacology and Experimental Therapeutics*.

[104] Wiegand F, Liao W, Busch C (1999). Respiratory chain inhibition induces tolerance to focal cerebral ischemia. *Journal of Cerebral Blood Flow and Metabolism*.

[105] Zhang Y, Lipton P (1999). Cytosolic Ca2+ changes during in vitro ischemia in rat hippocampal slices: major roles for glutamate and Na+dependent Ca2+ release from mitochondria. *Journal of Neuroscience*.

[106] Chen Y, Tsai Y-H, Tseng S-H (2011). The potential of tetrandrine as a protective agent for ischemic stroke. *Molecules*.

[107] Agostinho P, Cunha RA, Oliveira C (2010). Neuroinflammation, oxidative stress and the pathogenesis of Alzheimer’s disease. *Current Pharmaceutical Design*.

[108] Stahel PF, Smith WR, Bruchis J, Rabb CH (2008). Peroxisome proliferator-activated receptors: “Key” regulators of neuroinflammation after traumatic brain injury. *PPAR Research*.

[109] Hwang J, Adamson C, Butler D, Janero DR, Makriyannis A, Bahr BA (2010). Enhancement of endocannabinoid signaling by fatty acid amide hydrolase inhibition: a neuroprotective therapeutic modality. *Life Sciences*.

[110] O’Sullivan SE, Kendall DA, Randall MD (2009). Time-dependent vascular effects of endocannabinoids mediated by peroxisome proliferator-activated receptor gamma (PPAR*γ* ). *PPAR Research*.

[111] Mach F, Montecucco F, Steffens S (2008). Cannabinoid receptors in acute and chronic complications of atherosclerosis. *British Journal of Pharmacology*.

[112] Pegorini S, Zani A, Braida D, Guerini-Rocco C, Sala M (2006). Vanilloid VR1 receptor is involved in rimonabant-induced neuroprotection. *British Journal of Pharmacology*.

[113] Avraham Y, Davidi N, Porat M (2010). Leptin reduces infarct size in association with enhanced expression of CB_2_, TRPV1, SIRT-1 and leptin receptor. *Current Neurovascular Research*.

[114] Panikashvili D, Mechoulam R, Beni SM, Alexandrovich A, Shohami E (2005). CB_1_ cannabinoid receptors are involved in neuroprotection via NF-*κ*B inhibition. *Journal of Cerebral Blood Flow and Metabolism*.

[115] Du J, Wang Q, Hu B (2010). Involvement of ERK 1/2 activation in electroacupuncture pretreatment via cannabinoid CB_1_ receptor in rats. *Brain Research*.

[116] Mathew RJ, Wilson WH, Turkington TG (2002). Time course of tetrahydrocannabinol-induced changes in regional cerebral blood flow measured with positron emission tomography. *Psychiatry Research - Neuroimaging*.

[117] Singh NN, Pan Y, Muengtaweeponsa S, Geller TJ, Cruz-Flores S Cannabis-related stroke: case series and review of literature.

[118] Trojak B, Leclerq S, Meille V (2011). Stroke with neuropsychiatric sequelae after cannabis use in a man: a case report. *Journal of Medical Case Reports*.

[119] Finsterer J, Christian P, Wolfgang K (2004). Occipital stroke shortly after cannabis consumption. *Clinical Neurology and Neurosurgery*.

[120] Lawson TM (1996). Stroke and transient ischaemic attacks in association with substance abuse in a young man. *Postgraduate Medical Journal*.

[121] Mateo I, Pinedo A, Gomez-Beldarrain M, Basterretxea JM, Garcia-Monco JC (2005). Recurrent stroke associated with cannabis use. *Journal of Neurology, Neurosurgery and Psychiatry*.

[122] Kosior DA, Filipiak KJ, Stolarz P, Opolski G (2001). Paroxysmal atrial fibrillation following marijuana intoxication: a two-case report of possible association. *International Journal of Cardiology*.

[123] Varga K, Lake KD, Huangfu D, Guyenet PG, Kunos G (1996). Mechanism of the hypotensive action of anandamide in anesthetized rats. *Hypertension*.

[124] Ellis EF, Moore SF, Willoughby KA (1995). Anandamide and Δ9-THC dilation of cerebral arterioles is blocked by indomethacin. *American Journal of Physiology*.

[125] Wolff V, Lauer V, Rouyer O (2011). Cannabis use, ischemic stroke, and multifocal intracranial vasoconstriction: a prospective study in 48 consecutive young patients. *Stroke*.

